# Identification of Factors Influencing Cumulative Long-Term Radiation Exposure in Patients Undergoing EVAR

**DOI:** 10.1155/2017/9763075

**Published:** 2017-11-09

**Authors:** G. Kalender, Milan Lisy, U. A. Stock, A. Endisch, A. Kornberger

**Affiliations:** ^1^Department of Vascular and Endovascular Surgery, DRK Hospital Berlin-Köpenick, Berlin, Germany; ^2^Department of Vascular and Endovascular Surgery, Krankenhaus Nordwest, Frankfurt, Germany; ^3^Department of Cardiac and Transplant Surgery, Royal Brompton & Harefield NHS Trust, Harefield, UK; ^4^Department of Vascular and Endovascular Surgery, Asklepios Hospital, Bad Tölz, Germany; ^5^Department of Vascular, Thoracic and Cardiovascular Surgery, Universitätsmedizin der Johannes Gutenberg-Universität Mainz, Mainz, Germany

## Abstract

Patients who undergo endovascular repair of aortic aneurysms (EVAR) require life-long surveillance because complications including, in particular, endoleaks, aneurysm rupture, and graft dislocation are diagnosed in a certain share of the patient population and may occur at any time after the original procedure. Radiation exposure in patients undergoing EVAR and post-EVAR surveillance has been investigated by previous authors. Arriving at realistic exposure data is essential because radiation doses resulting from CT were shown to be not irrelevant. Efforts directed at identification of factors impacting the level of radiation exposure in both the course of the EVAR procedure and post-EVAR endovascular interventions and CTAs are warranted as potentially modifiable factors may offer opportunities to reduce the radiation. In the light of the risks found to be associated with radiation exposure and considering the findings above, those involved in EVAR and post-EVAR surveillance should aim at optimal dose management.

## 1. Introduction

Patients undergoing endovascular repair of aortic aneurysms (EVAR) require life-long surveillance to detect potential complications such as endoleaks, aneurysm rupture, and graft dislocation [1–4]. As far as the imaging modalities and the follow-up schedules clinical practice differs [5, 6]. Usually, however it is a period of more frequent examinations followed by yearly checks unless pathology requires closer surveillance [1, 3, 7]. The method most frequently applied is computed tomography angiography (CTA) [1, 5, 8], even though radiation exposure is associated with a risk of malignancy and CTA requires far more exposure to radiation than other radiological examination methods [9].

Radiation exposure in patients undergoing EVAR and post-EVAR surveillance has been investigated by previous authors who mostly used institutional mean values or radiation exposure levels for different types of examinations derived from literature or extrapolated from a small number of measurements performed [10–15]. Our study differs from existing literature in that we individually calculated an effective dose (ED) for each procedure and examination performed in our study population over a period of six and a half years in order not only to arrive at realistic exposure and risk data, but also to gain an insight into the factors that exert an influence on the level of radiation exposure.

## 2. Material and Methods

### 2.1. Patients, Procedures, and Surveillance Schedule

All patients who underwent endovascular repair of abdominal aortic aneurysms between January 2004 and May 2010 were retrospectively identified, and the periods for which they were followed up were determined. Those with follow-up periods shorter than 6 months were excluded. Electronic and paper patient records, stored angiography, and CTA protocols, as well as DICOM header data, were available as data sources. Supplementary telephone inquiries were made to follow up patients who had not presented for examination for more than one year. For the purposes of the present study, each patient was observed for an observation period commencing at the date of the preoperative CTA and ending on 10 August 2011 unless the patient died or post-EVAR surveillance was continued by another hospital/physician or was switched to a different imaging modality before this date. In cases where the exact date of the preoperative CTA could not be ascertained or no evaluable preoperative CTA was available, the date of the EVAR was substituted for the beginning of the observation period.

EVAR procedures were performed jointly by a senior vascular surgeon and a senior radiologist in an angiography suite, after obtaining patient consent on the basis of comprehensive information provided not only on procedural details and risks, but also on radiation exposure and the need for life-long post-EVAR surveillance. CTAs were performed by our hospital's Department of Radiology unless patients preferred to have CTAs performed by local radiologists.

The following standards were applied for pre- and post-EVAR CTAs:

- Routine protocol: CTA of the abdomen and pelvis, with concomitant scanning of adjoining regions (thorax and/or lower extremities) in cases where pathology extending beyond the abdomen and pelvis is present.
- Minimum requirement for preoperative CTA: biphasic protocol comprising native scanning and the arterial phase of enhancement.
- Initial post-EVAR CTA: triphasic protocol comprising native scanning, the arterial phase of enhancement, and a late phase after 2-3 minutes. A four-phase protocol may be required in special cases, for example, where identification of an endoleak requires an additional effort.
- Subsequent CTAs: mono- or multiphase protocols as considered appropriate.

Follow-up CTAs were performed 3, 6, and 12 months after the EVAR procedure. After the first year, surveillance was reduced to yearly CTAs unless more frequent follow-ups were required by presence of pathology. Patients in whom no pathology was identified and in whom renal impairment, allergy to the contrast agent, or other circumstances warranted longer follow-up intervals were switched to individually designed surveillance schedules on a case-to-case basis. For each patient, we determined how many of the follow-ups required by the surveillance schedule were actually implemented. The target number of follow-ups, that is, 100% follow-up compliance, was defined as 3 CTAs during the first year and 1 CTA during each subsequent year. In patients who underwent additional CTAs, the follow-up compliance was also considered to be 100% as allocation of compliance values exceeding 100% would have masked noncompliance within the study population. Patients who underwent at least 75% of the follow-ups required according to the schedule were considered to be “compliant” while those who underwent less than 75% of the follow-ups scheduled were classified as “noncompliant.”

### 2.2. Determination of Radiation Exposure

The radiation exposure resulting from the EVAR procedure and subsequent endovascular interventions was determined by using the dose area product (DAP) (Gy × cm^2^) indicated in the intervention protocol. The DAP was multiplied with a weighting factor of 0.145 [10, 12], with the area exposed to fluoroscopy extending from the origins of the visceral arteries to the puncture sites in the femoral arteries.

The ED from each CTA was determined using CT-EXPO2.0 [16, 17] (version as of Jan 2011), which allows gender-specific dose calculation according to latest standards (ICRP 103, 2007) for any existing scanner type and anatomic region or scan range. A pitch of 1 was assumed where total collimation and table travel could not be ascertained from the data available. The manual scan range selection function offered by the software was applied when complex examination protocols were used. Where a fourth phase was implemented, which was the case in few examinations only, it was added to the third, venous phase for reasons of simplification. All scanning parameters were entered separately for each phase. From the results output, the ED per phase and the total ED for the entire examination were used for further analysis including investigation of a number of factors suspected of impacting radiation exposure.

From the individual dose values obtained, we determined a cumulative ED for the first-year post-EVAR and the cumulative annual ED delivered during each subsequent year. In a standard patient, the first-year cumulative ED comprised the preoperative CTA, the EVAR implantation procedure, the initial post-EVAR CTA, and the 6- and 12-month CTA follow-ups. The mean annual ED for the subsequent years was only calculated for patients followed up for at least two years and in a standard patient comprised one CTA per year. However, calculation was not performed based on the examinations prescribed by the surveillance schedule but included only those actually performed. Extra CTAs exceeding the follow-ups provided for by the surveillance schedule as well as secondary endovascular procedures performed during the observation period were included into the calculation. From the values thus obtained, we extrapolated EDs for 5- and 10-year surveillance periods to allow comparison between our data and the findings of previous authors.

## 3. Statistical Methods

All data was compiled in an Excel file (Microsoft Corp.). Statistical analysis was performed using the statistics software “R.” Mean values and standard deviations were indicated for normally or nearly normally distributed data, while nonnormally distributed data were reported in the form of median values, interquartile ranges (IQR), and minimum and maximum values. The Kruskal-Wallis test, the Wilcoxon test, and Student's *t*-test were applied as appropriate to determine the impact of a variety of factors on the ED, with *p* values < 0.05 considered to be statistically significant. Additionally, Pearson correlation coefficients were determined to measure the strengths of relationships.

## 4. Results

The study population comprised 59 patients. Patient characteristics as well as information relating to complications and additional procedures are summarized in Table 1.

The EVAR procedures included in the present study were performed between 13 January 2004 and 05 May 2010. Patients were followed up for a median observation period of 27 months (minimum 6 months, maximum 91 months). Of the follow-ups required according to the surveillance schedule during the first-year post-EVAR, only 68.3% were actually implemented. Of those required from the second year onwards, an average of 70% was actually performed.

The total number of CTAs included into the analysis was 251, with each patient undergoing a mean of 4.3 examinations (range 2–9) during the observation period and evaluable preoperative CTA's performed a mean of 34 days before the EVAR procedure available for 47 patients (79.7%). The initial post-EVAR CTA was performed a mean of 12 days post-EVAR. The median ED from all CTAs was 24.5 mSv (Table 2), with a triphasic protocol applied in 184 (73.3%) of the examinations. The shares of triphasic examinations in the preoperative CTAs, the initial post-EVAR CTAs, and yearly follow-up CTAs were 66%, 86.4%, and 70.3%, respectively. The median EDs per phase were 4.5, 12.7, and 9.6 mSv for the native, arterial, and late venous phases (Table 2).

Application of the Wilcoxon test yielded a statistically significant (*p* < 0.001) difference between the median EDs from mono- or biphasic CTAs (17.6 mSv) and triphasic CTAs (26.6 mSv). In 32% of the CTAs, the thorax was scanned in addition to the abdomen and pelvis. This extension of the scanned region led to a statistically significant increase of the ED (*p* = 0.012; Wilcoxon test) from a median ED of 23.8 mSv for scanning of the pelvis/abdomen to a median ED of 26.4 mSv for scanning of the pelvis/abdomen and thorax.

**Figure 1:**
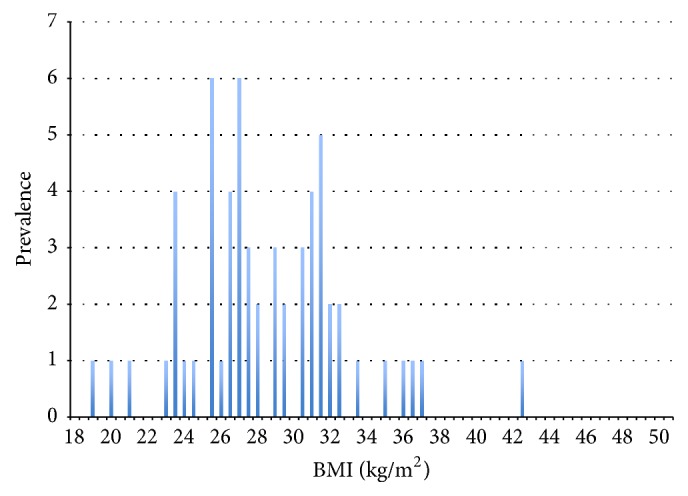
Distribution of the study population over the WHO body mass classes, with dotted lines separating “underweight/normal weight,” “overweight,” and “obese.”

**Figure 2:**
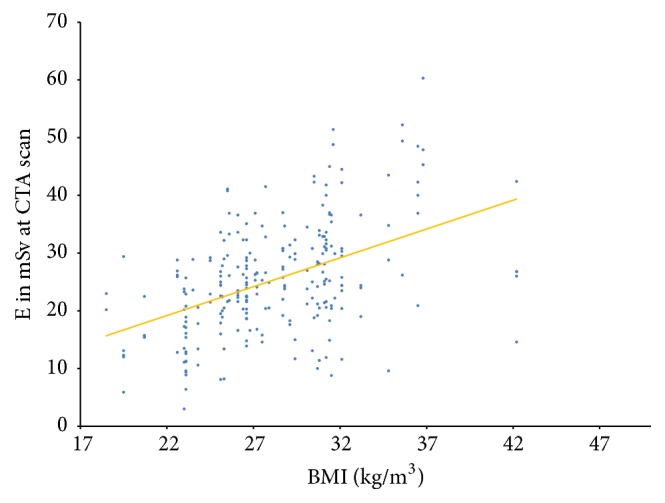
Correlation between ED from CTA [in mSv] and body mass index [in kg/m2].

The distribution of the study population over the WHO BMI classes is shown in Figure 1. Statistical analysis using the Kruskal-Wallis test additionally showed a significant positive correlation between the ED from CTA and the body mass index (*p* < 0.001, Pearson correlation coefficient 0.464) (Figure 2).

The CTAs were performed on ten different scanner types as follows:

  Siemens Somatom Definition  *n* = 165
  Siemens Somatom Sensation 16  *n* = 6
  Siemens Somatom Sensation 10  *n* = 2
  Siemens Somatom Sensation 64  *n* = 23
  Siemens Somatom Sensation Open  *n* = 9
  Siemens Somatom Volume Zoom  *n* = 1
  Siemens Somatom Definition AS+  *n* = 7
  Siemens Somatom Emotion 6  *n* = 3
  Siemens Somatom Definition Flash  *n* = 34
  ToshibaXpress GX  *n* = 1

**Figure 3:**
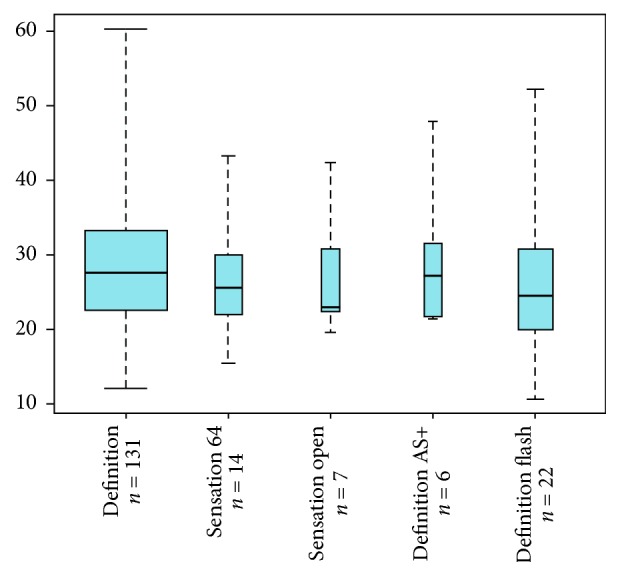
EDs [in mSv] from triphasic CTA scans performed on the 5 scanner types employed most frequently.

Comparison between the EDs for triphasic scans performed using the five scanner types employed most frequently, that is, for more than 5 CTAs, showed no significant differences (*p* = 0.624; Kruskal-Wallis test) (Figure 3). Mean values were between 27.2 and 29.4 mSv.

**Figure 4:**
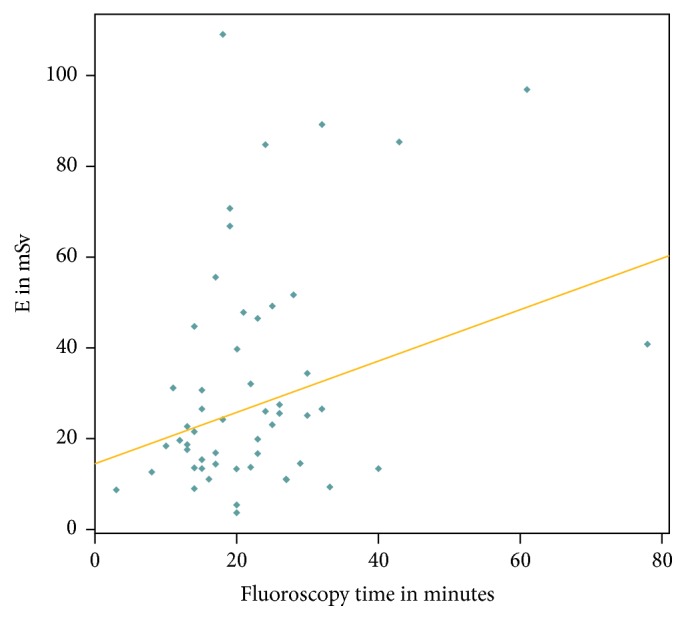
Correlation between fluoroscopy time [in minutes] and ED [in mSv] from the EVAR procedure.

The median ED and the mean fluoroscopy time during the EVAR procedure were 23 mSv and 22.2 ± 12.3 minutes, respectively. A weak positive correlation was found between the fluoroscopy time and the ED from EVAR (Pearson correlation coefficient 0.36) (Figure 4).

**Figure 5:**
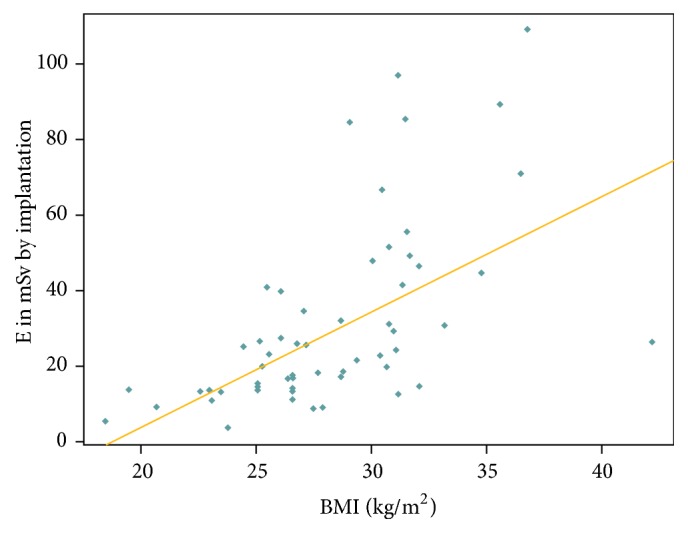
Correlation between body mass index [in kg/m2] and ED [in mSv] from the EVAR procedure.

A statistically significant positive correlation (Pearson correlation coefficient 0.591) (Figure 5) was identified between the ED from the EVAR and the body mass index.

This was confirmed by applying the Kruskal-Wallis test, which yielded a significant positive correlation between ED and body mass index (*p* < 0.001), to the median EDs from the EVAR procedures performed in patients classified, according to the WHO, as underweight/normal weight (<25 kg/m2, median ED 13.4 mSv), overweight (25–30 kg/m2, median ED 18.5 mSv), and obese (>30 kg/m2, median ED 45.7 mSv).

**Figure 6:**
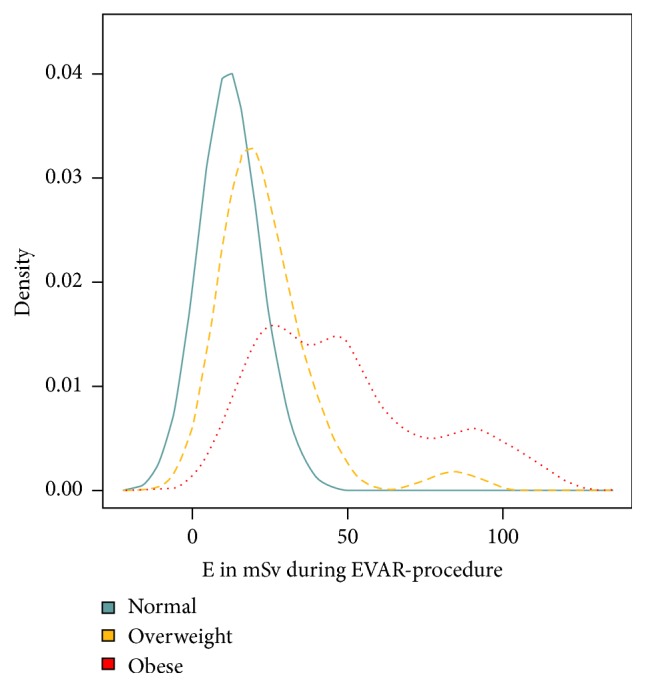
Density curves for the ED delivered to the three WHO BMI categories during EVAR.

**Figure 7:**
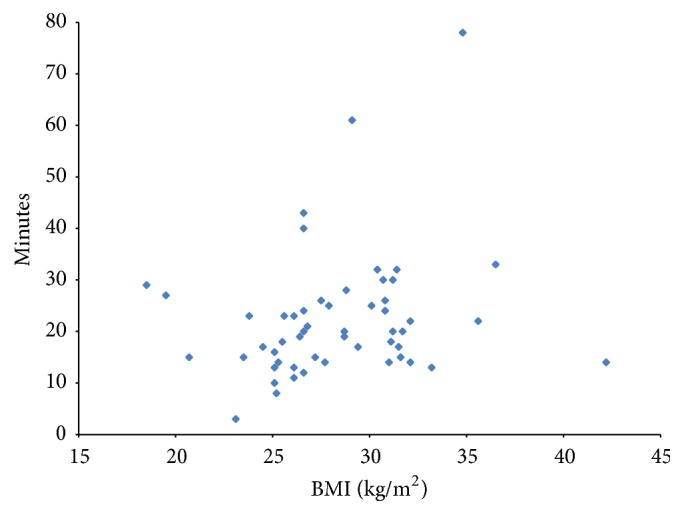
No correlation is identified between the fluoroscopy time and the body mass index.

This is also illustrated by the density curves for the ED delivered to the three WHO BMI classes during the EVAR procedure (Figure 6). With a view to excluding that higher radiation exposure in overweight and obese patients resulted from greater complexity of the EVAR procedures in this particular subset of the EVAR population, the fluoroscopy time was applied as a surrogate for the complexity of the procedure. However, there was no correlation between the fluoroscopy time and the body mass index (Pearson correlation coefficient −0.11) (Figure 7).

The median DAP and ED determined for the 11 secondary endovascular procedures performed in 9 patients, consisting of fibrinolysis, angioplasty, and/or stent angioplasty for graft limb occlusion in 5 cases, embolization of endoleaks in 3 cases, and stent angioplasty in one case each of renal artery stenosis and iliac artery stenosis, were 143.4 Gycm^2^ and 20.8 mSv, respectively.

The minimum and maximum cumulative EDs for the entire observation period were 55 mSv and 310 mSv, respectively. The mean cumulative ED for the first-year post-EVAR amounted to 109 ± 43.7 mSv, and the ED delivered during each subsequent year was 16 mSv /year (IQR 16.3). The largest share of the ED both during the first-year post-EVAR (69.2%) and during subsequent years (94.3%) resulted from CTAs. The initial EVAR procedure accounted for 27.7% of the first-year cumulative ED, while 3.1% and 5.7% of the first-year and subsequent year cumulative EDs resulted from secondary endovascular procedures.

**Figure 8:**
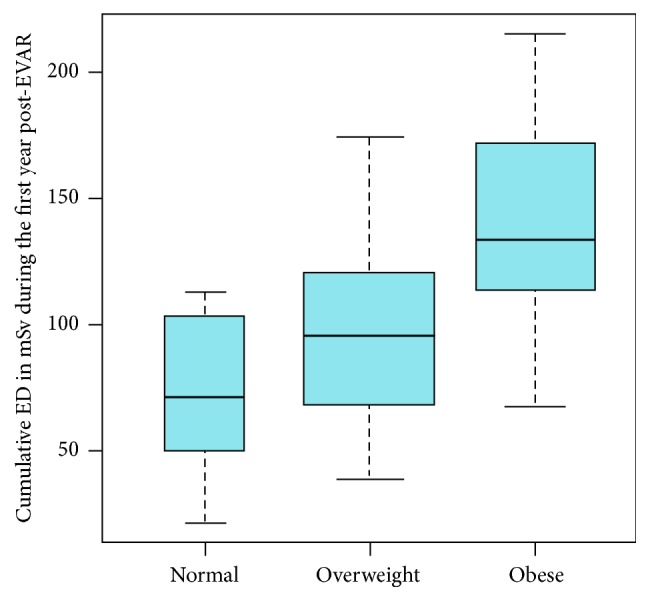
Cumulative ED [in mSv] during the first-year post-EVAR, shown by body mass index categories, with the width of the box plots adjusted to the number of patients per category.

Analysis of the cumulative ED during the first-year post-EVAR by body mass index shows a clear increase of radiation exposure with increasing body mass index, with the *t*-test showing statistically significant differences (*p* < 0.001), apart from the comparison between normal and overweight patients (*p* = 0.058), between the mean values determined for the body mass classes (Figure 8).

With regard to implementation of the follow-ups prescribed by the surveillance schedule and using a cut-off value of 75%, 39% of our study population were noncompliant in that they underwent less than 75% of the follow-ups. Analysis of the mean cumulative ED during the first-year post-EVAR using the* t*-test, however, showed no statistically significant difference (*p* = 0.373) between compliant (114 mSv) and noncompliant (103.3 mSv) patients. This was confirmed for the cumulative ED during the subsequent years by application of the Wilcoxon test, which showed no statistically significant differences (*p* = 0.054) between compliant and noncompliant patients either.

The mean ED during the first-year post-EVAR also did not differ significantly (*t*-test,* p* = 0.352) between patients with endoleaks (117.2 mSv) and patients without endoleaks (105.4 mSv). Significant differences between the cumulative EDs determined in patients with and without endoleaks were not identified for the subsequent years either (*p* = 0.085, Wilcoxon test) even though patients with endoleaks underwent extra examinations.

## 5. Discussion

CTA accounted for the major share of both the cumulative first-year post-EVAR ED (69.2%) and the cumulative subsequent yearly ED (94.3%), while the EVAR procedure (27.7% of the first-year cumulative ED) and secondary endovascular procedures (3.1% of the first-year cumulative ED and 5.7% of the subsequent yearly ED) contributed only little to the total ED delivered to EVAR patients in our study. This may be assumed to be fairly the same in all programs consisting of an EVAR procedure that is followed by life-long CTA surveillance. Therefore, factors influencing the radiation dose delivered by CTA are of particular interest when it comes to reducing radiation exposure and the risk of radiation-induced pathology in patients treated by EVAR.

Accordingly, reducing the number of CTAs performed appears to be the most obvious way to reduce the radiation dose delivered. However, comparison between the cumulative radiation doses delivered to compliant (i.e., implementation of ≥75% of the CTAs required) versus noncompliant (i.e., implementation of <75% of the CTAs required) patients in our patient cohort yielded no statistically significant difference. This finding is even more relevant when taking into account the fact that patients who underwent more than the number of CTAs prescribed by the surveillance schedule, for example, where pathology was present and needed closer surveillance, were also included into the compliant group, thus increasing the cumulative ED of the compliant group in comparison with the noncompliant group. This finding may be interpreted as suggesting that a moderate reduction of the number of CTAs, for example, by switching from yearly to longer intervals after a certain surveillance period or by introducing surveillance protocols where CTA and imaging techniques not involving ionizing radiation are scheduled alternately, may not yield a tremendous benefit with a view to exposure reduction. Alternative methods such as contrast-enhanced ultrasound (CEUS), magnetic resonance imaging (MRI), and implantable wireless aneurysm sac pressure sensors have been investigated and were suggested to be applied in post-EVAR surveillance, but completely banning CTA from post-EVAR follow-ups is currently not generally considered advisable [18–22].

In addition to reducing the number of CTAs, reducing the number of phases per CTA may be considered. A review of literature in fact shows that the value of the individual phases in post-EVAR CTA surveillance is an ongoing subject of dispute [23–25]. Comparison between our own findings and radiation exposure levels reported by previous authors in the framework of an earlier study has shown that our ED per CTA (24 mSv) was not only among the highest but also that a large proportion of our CTAs comprised three phases. The authors of previous studies, in contrast, had reported considerable shares of mono- and biphasic CTAs, with Kalef-Ezra et al. reporting a reduction of the ED delivered through the 3 CTAs performed during the first year to a total of 33 mSv simply by eliminating the unenhanced phase [10–12]. Following the same line, White and Macdonald argued that a reduction of radiation exposure by 44% could be achieved by reducing the surveillance protocol to biphasic CTA comprising a native and a late venous phase only, without incurring a significant loss of information in terms of endoleak identification [15]. Similar effects could be confirmed within our study population, where a statistically significant difference was determined between the ED from mono- or biphasic CTAs (17.6 mSv), on the one hand, and triphasic CTAs (26.6 mSv), on the other.

Schabel et al., investigating patients who underwent endovascular repair of infrarenal aortic aneurysms, adopted a modified approach by conducting surveillance by means of a nonenhanced CT scan followed by volumetric analysis in patients in whom the initial postinterventional follow-up had shown no pathology. Enhanced CT was subsequently performed only in patients in whom the volumetric analysis had shown an enlargement of the aneurysm sac of >2% in comparison with the most recent prior scan. The reduction in radiation exposure achieved by application of this protocol was reported to be 69–82% in comparison with traditional triphasic protocols consisting of nonenhanced scanning followed by contrast-enhanced and delayed postcontrast phases and 57–72% in comparison with protocols comprising unenhanced and contrast-enhanced scanning only [26].

Two further potentially modifiable factors examined in the context of the present study were the scanner type used and the extension of the region scanned. While the former was shown to have exerted no relevant influence on radiation exposure, a statistically significant effect was shown for the latter by comparing the EDs for regular scanning of the abdomen/pelvic region, on the one hand, and extended scans including the chest, on the other. The clinical relevance of the difference between median EDs of 23.8 mSv for scanning of the pelvis/abdomen and 26.4 mSv for scanning of abdomen, pelvis, and thorax, however, is probably limited. But as concomitant scanning of the chest was performed in 32% of the CTAs, it may be assumed that these findings reflect day-to-day clinical practice in that extended scanning may in some cases have been ordered routinely or indiscriminately rather than being based on actual or suspected thoracic pathology. Where this is the case, closer attention to the patient's actual imaging needs and more judicious ordering of CT scans could contribute to reducing the radiation dose delivered to patients in the long term.

New iteration algorithms such as SAFIRE (Sinogram Affirmed Iterative Reconstruction) may be applied and were reported to be able to achieve a dose reduction of up to 50% in post-EVAR surveillance by iterative image optimization [27].

In our study population, but also in those investigated, for example, by Weiss et al. [14] and Kalef-Ezra et al. [10], the patient's body mass index exerted a relevant influence on the radiation burden. Uppot [28] retrospectively analyzed 92 patients who underwent EVAR and similarly found that a body mass exceeding 25, along with tortuosity of the iliac arteries and short aneurysm necks, was a main factor contributing to high radiation doses being acquired by patients during EVAR. These findings are not surprising, considering that imaging in obese patients is known to pose particular challenges [29–31] and was therefore also referred to, for example, in the Society of Interventional Radiology in its Guidelines for Patient Radiation Dose Management [32].

We applied the BMI classification proposed by the WHO and were able to identify, in particular, a statistically significant positive correlation between the BMI and the ED from CTA. Additionally, we were also able to demonstrate that the mean ED delivered during the first-year post-EVAR, that is, including the EVAR procedure, was significantly higher in obese than in overweight patients, while a difference between underweight/normal weight and overweight patients was also identified but not statistically significant. As it may be argued, with regard to the EVAR procedure, that the anatomy of obese patients may lead to greater complexity of the procedure and, hence, to a higher radiation exposure, we used the fluoroscopy time as a surrogate for the complexity of the procedure and found that no statistically significant positive correlation exists between the BMI and the fluoroscopy time.

As alternative imaging methods such as ultrasound, in particular, frequently yield poor results in obese patients, too, the opportunities to reduce radiation exposure in this particular subset of patients are limited to providing them with adequate information and lengthening the intervals between CTA follow-ups as well as limiting the number of scans per CTA where appropriate. Weight loss should of course be recommended, especially as obesity was recently found to be an independent predictor of outcome after endovascular abdominal aortic aneurysm repair [33].

With regard to the EVAR procedure, our data showed a trend towards a lower radiation exposure during the last 12–18 months of the study period, which may be interpreted as reflecting a learning curve or operator dependence of the ED and representing a potentially modifiable factor impacting the radiation dose delivered to patients not only during the EVAR procedure but also during any secondary endovascular procedures performed. In addition to factors relating to the level of experience available at a given institution, to operators, learning curves and the continuity of team composition, an influence on the ED delivered during the EVAR procedure is of course also exerted by the equipment used. While we were able to show that the type of scanner used to implement CTAs does not give rise to statistically significant differences between the EDs delivered, comparison between the values published by previous authors showed that the highest EDs resulting from the EVAR procedure were reported by institutions where EVAR was performed in angiography suites, for example, 354 Gycm^2^ reported by Blaszak et al. [33] or 160 Gycm^2^ determined in our study population, while values reported by investigators using mobile C arms were lower. This is confirmed by Geijer et al. [12] and Fossaceca et al. [34] and most likely explained by the higher performance of the DSA systems used in angiographic suites.

Interestingly, presence of endoleaks was not associated with a statistically significant increase in radiation exposure even though one would expect additional radiation exposure not only to arise from shorter intervals between follow-ups but also to result from endovascular correction procedures in patients with endoleaks. However, all primary endoleaks, diagnosed in 19 of 59 patients, were type II endoleaks and resolved spontaneously within the first year in 81% of the 12 patients in whom spontaneous resolution was observed and were successfully treated by endovascular intervention in 3 further patients, thus requiring no extra follow-ups after elimination of the pathology. Of the secondary endoleaks, a relevant influence on the cumulative ED of the study population was probably exerted only by the case of a type II endoleak diagnosed 15 weeks after EVAR in which closer surveillance was required while the aneurysm sac was shrinking. The patient with a type II secondary endoleak diagnosed 7 weeks after EVAR died for nonaneurysm-related causes before undergoing further examinations or interventions, and the patient with a type I endoleak who was surgically treated for graft dislocation did not relevantly increase the collective radiation burden either because the pathology was removed rather than setting the surveillance schedule to shorter follow-up intervals. The endovascular procedures performed in patients with endoleaks similarly did not contribute exorbitantly to the total radiation burden as all secondary endovascular procedures performed accounted to only 3.1% and 5.7% of the first year and subsequent yearly EDs, respectively.

The mode of patient presentation was repeatedly shown to be another predictor for the degree of radiation exposure, with higher radiation exposure values reported in patients undergoing EVAR on an emergency than on an elective basis [35]. It may, in fact, be assumed that contrast and fluoroscopy are more liberally applied in a situation where a patient with a ruptured aortic aneurysm is in need of rapid hemodynamic stabilization than in an elective setting where care is taken to keep the radiation burden as low as possible. Badger et al., referring to aortoiliac stent-grafts, additionally argued that a different technique may be preferred in emergent cases to obtain hemodynamic control as quickly as possible [35]. In our patient cohort, however, the impact of the mode of patient presentation was not analyzed as the number of emergencies was too small.

## 6. Conclusions

In the light of the risks found to be associated with radiation exposure and considering the findings above, those involved in EVAR and post-EVAR surveillance should aim at optimal dose management. This may include reducing the number of scanning phases, including alternative methods into post-EVAR follow-up, and modifying surveillance schedules on a case-to-case basis taking patient age, presence, or absence of pathology, as well as the duration of pathology-free survival into account. In the setting of the EVAR procedure as such, availability of well-trained personnel and establishment of treatment routines may prevent unnecessarily high radiation exposure. Overall, however, the number of modifiable factors impacting the radiation burden in patients undergoing EVAR is small, while the majority of factors including the body mass index, the patient's emergency status, pathologies such as endoleaks requiring closer monitoring, and the equipment available at a given institution are largely not amenable to modification.

## Figures and Tables

**Table 1:** Patient characteristics and details on occurrences during the observation period.

Patient characteristics, complications, and secondary surgical/endovascular interventions		
Total number of patients	59	
Male versus female patients	55 (93.2%)	4 (6.8%)
Emergent versus elective EVAR	3 (5%)	56 (95%)
Mean age at the date of implantation	70 yrs	(41–83 yrs)
Mean body mass index	28 ± 4.3 kg/m2.	
Observation period terminated by non-EVAR related death	8 (13.6%)	
Lost to follow-up	2 (3.4%)	
Compliance versus noncompliance with the surveillance schedule	36 (61%)	23 (39%)
Open surgical correction procedures	2 (3.4%)	
(i) Aortorenal bypass grafting for renal artery occlusion	1 (1.7%)	
(ii) Explantation of a migrated stent graft and implantation of a bifurcated vascular graft	1 (1.7%)	
Long-term corticosteroid treatment for suspected periprosthetic inflammatory reaction	1 (1.7%)	
Endoleak (median persistence 222 days (IQR 66–787 days))	22 (37.3%)	
Primary endoleak (i.e., occurring within 30 days from EVAR), all classified as type II	19 (32.2%)	
(i) Permanent	4 (21.0%)	
(ii) Treated by endovascular intervention	3 (15.8%)	
(iii) Spontaneous resolution (81% resolved during the first year post-EVAR)	12 (63.2%)	
Secondary endoleak (i.e., occurring more than 30 days from EVAR)	3 (5%)	
(i) Type II endoleak diagnosed 7 weeks after EVAR, death for nonaneurysm related causes	1 (1.7%)	
(ii) Type II endoleak diagnosed 15 weeks after EVAR, spontaneous shrinkage of the aneurysm sac, no interventional or surgical correction but close surveillance	1 (1.7%)	
(iii) Type I endoleak resulting from graft dislocation 3 years after EVAR, treated by open surgical repair	1 (1.7%)	
Secondary endovascular procedures	11 in 9 patients	
(i) Minimum/maximum period from EVAR to secondary endovascular procedure	1 day/3.3 years	
(ii) Fibrinolysis, angioplasty, and/or stent angioplasty for graft limb occlusion	5/11	
(iii) Embolization of endoleaks	3/11	
(iv) Stent angioplasty for renal artery stenosis	2/11	
(v) Stent angioplasty for iliac artery stenosis	1/11	

**Table 2:** Effective doses from CTA and interventional procedures [in mSv].

Effective doses from CTA and endovascular procedures [in mSv]						
	Min. ED (mSv)	1st quartile (mSv)	Median ED (mSv)	3rd quartile (mSv)	Max. ED (mSv)	*p*
CTA	3.0	19.7	24.5	30.3	60.3	
Native phase	0.9	2.9	4.5	5.6	18.8	
Arterial phase	1.9	9.6	12.7	15.7	27.3	
Late venous phase	2.6	7.7	09.6	12.4	23.4	

Mono- or biphasic CTA versus			17.6			
Triphasic CTA			26.6			<0.001

CTA pelvis/abdomen versus			23.8			
CTA pelvis/abdomen/thorax			26.4			0.012

EVAR	3.6	14.3	23.2	40.8	109.2	

EVAR in normal weight patient versus			13.4			
EVAR in overweight patient versus			18.5			
EVAR in obese patient			45.7			<0.001

Secondary endovascular procedures	2.6	13.6	20.8	25.5	60.7	

## Additional Points

*Limitations*. The main limitation of the present study is the moderate number of patients included. This moderate number of patients, however, allowed all procedures and examinations requiring application of ionizing radiation performed in each of the patients during the observation period to be included separately, with separate calculation of radiation doses for each individual examination and procedure. As exposure data from procedures involving ionizing radiation is meticulously recorded and stored at our hospital, the retrospective nature of the study did not cause a relevant loss of information apart from minor shortcomings in a small number of cases in which CTAs were performed by local radiologists and where certain information was missing and could not be ascertained retrospectively.
